# Dynamic spatiotemporal beams that combine two independent and controllable orbital-angular-momenta using multiple optical-frequency-comb lines

**DOI:** 10.1038/s41467-020-17805-1

**Published:** 2020-08-14

**Authors:** Zhe Zhao, Hao Song, Runzhou Zhang, Kai Pang, Cong Liu, Haoqian Song, Ahmed Almaiman, Karapet Manukyan, Huibin Zhou, Brittany Lynn, Robert W. Boyd, Moshe Tur, Alan E. Willner

**Affiliations:** 1 Department of Electrical Engineering, University of Southern California, Los Angeles, CA 90089 USA; 2 King Saud University, Riyadh, 11362 Saudi Arabia; 3 Naval Information Warfare Center Pacific, San Diego, CA 92152 USA; 4 Department of Physics, University of Ottawa, Ottawa, ON Canada; 5 The Institute of Optics, University of Rochester, Rochester, NY 14627 USA; 6 School of Electrical Engineering, Tel Aviv University, Ramat Aviv, 69978 Israel

**Keywords:** Optical physics, Optical techniques, Frequency combs

## Abstract

Novel forms of beam generation and propagation based on orbital angular momentum (OAM) have recently gained significant interest. In terms of changes in time, OAM can be manifest at a given distance in different forms, including: (1) a Gaussian-like beam dot that revolves around a central axis, and (2) a Laguerre-Gaussian ($LG_{\ell ,p}$) beam with a helical phasefront rotating around its own beam center. Here we explore the generation of dynamic spatiotemporal beams that combine these two forms of orbital-angular-momenta by coherently adding multiple frequency comb lines. Each line carries a superposition of multiple $LG_{\ell ,p}$ modes such that each line is composed of a different $\ell$ value and multiple *p* values. We simulate the generated beams and find that the following can be achieved: (a) mode purity up to 99%, and (b) control of the helical phasefront from 2*π*-6*π* and the revolving speed from 0.2–0.6 THz. This approach might be useful for generating spatiotemporal beams with even more sophisticated dynamic properties.

## Abstract (web summary)

Orbital angular momentum takes several forms in structured light beams. Here, the authors demonstrate control of dynamic spatiotemporal beams combining two forms of orbital angular momenta, by coherently adding frequency comb lines.

## Introduction

**Fig. 1:**
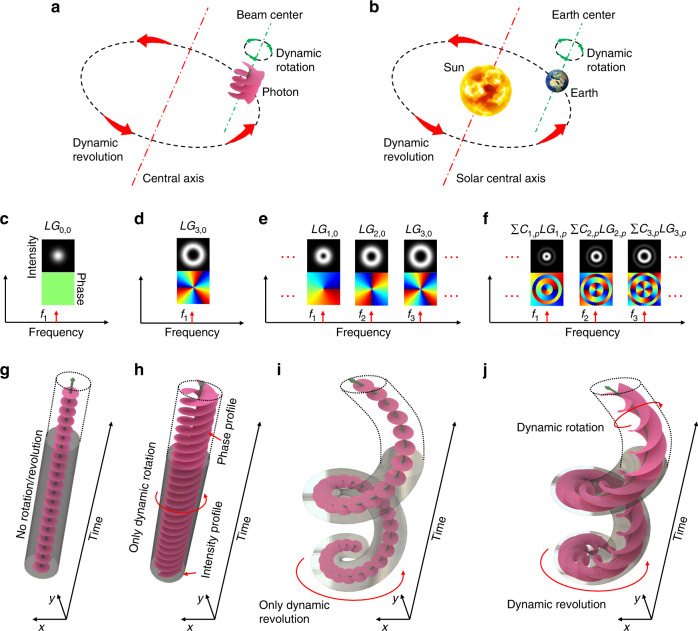
Generating a spatiotemporal beam exhibiting both dynamic rotation and revolution. **a**, **b** Illustration of a light beam that dynamically rotates around its beam center and revolves around another central axis; this is analogous to the earth orbiting around the sun, exhibiting both rotation around its Earth center and revolution around the solar central axis. **c**, **g** A Gaussian beam on a single frequency exhibits no dynamic rotation/revolution. **d**, **h** An *LG*_3,0_ beam on a single frequency exbibits only dynamic rotation. **e**, **i** Using multiple frequency comb lines, in which each carries an $LG_{\ell ,p}$ mode with a different $\ell$ value and the same *p* value, to generate a Gaussian-like beam dot exhibiting only revolution around a central axis. **f**, **j** Using multiple frequency comb lines, in which each carries a superposition of multiple $LG_{\ell ,p}$ modes with one different $\ell$ value and multiple *p* values, to generate an *LG*_3,0_ beam exhibiting both dynamic rotation and revolution.

Structured light has recently gained increased interest in that it can accommodate the production of uniquely propagating beams of light^1–5^. One particularly interesting aspect is the ability of a structured beam to carry orbital angular momentum (OAM)^6–11^. One form of momentum is a simple Gaussian beam dot that can rotate in a circular fashion as it propagates, illuminating a ring shape^12–14^; this OAM is similar to revolution around a central axis. A second form of momentum is a subset of Laguerre–Gaussian ($LG_{\ell ,p}$) beams in which the phasefront twists in the azimuthal direction as it propagates^15,16^. The amount of OAM ($\ell$) is the number of 2*π* azimuthal phase changes, and *p* + 1 is the number of concentric rings on the intensity cross section for $\ell \,\neq \, 0$. The beam rotates around its own beam center with a ring-like vortex intensity profile (Fig. 1d, h). This second type of OAM is similar to rotation. Indeed, the earth propagating around the sun exhibits both rotation around its own Earth center and revolution around a solar central axis^17^.

These two manifestations of momentum can occur in space during propagation, but yet the beam’s intensity will appear static at any given point of propagation distance^18–22^. This scenario can be made more complex by enabling the generation and propagation of a dynamic spatiotemporal beam, such that the beam simultaneously revolves and rotates in the *x*–*y* plane in time at a given propagation distance *z*. Prior art has produced novel beams by combining different modes not only on the same frequency^18–22^ but also on different frequencies^12–14,23–27^. This ability to produce different modes on different frequencies can be achieved by the use of optical-frequency combs, which have recently undergone much advancement^28^.

**Fig. 2:**
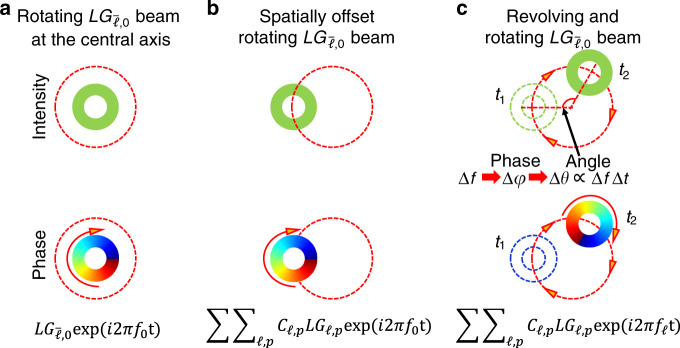
Coherent interference of multiple frequency comb lines leading to the dynamic rotation and revolution. **a** $LG_{\bar \ell ,0}$ beam on a single frequency whose center located at the central axis. **b** The interferogram of multiple $LG_{\ell ,p}$ modes with multiple $\ell$ values and *p* values carried by a single frequency line. It produces an $LG_{\bar \ell ,0}$ beam whose beam center is offset from the original central axis. **c** Combining multiple frequency comb lines with each carrying a superposition of multiple $LG_{\ell ,p}$ modes (a different $\ell$ value and multiple *p* values). Δ*φ*: time-variant relative phase delay between neighboring $LG_{\ell ,p}$ modes, leading the generated light beam to dynamically revolve around the central axis; Δ*f*: frequency spacing; Δ*θ*: revolving angle; Δ*t*: *t*_1_ – *t*_2_ is the temporal delay.

Specifically, it has been previously shown that a light beam can be created to exhibit unique dynamic features^12–14,23–27,29–35^, including the following examples: (a) a Gaussian-like beam dot that exhibits dynamic circular revolution at a given propagation distance by combining multiple frequency lines in which each line carries a different $LG_{\ell ,p}$ mode (different $\ell$ but same *p*)^12–14^ (Fig. 1e, i); (b) a Gaussian-like beam dot formed from multiple Hermite–Gaussian modes, each at a different frequency, such that the dot can dynamically move up-and-down in a linear fashion at a given propagation distance^23^; (c) a light beam created by a pair of $LG_{\ell ,p}$ modes with different $\ell$ and *p* values at two different frequencies, such that it exhibits dynamic rotation around its center but no dynamic revolution around another axis at a given propagation distance^26^; (d) a combination of multiple frequency lines, each of which carries one $LG_{\ell ,p}$ mode with a different pair of indices ($\ell ,p$) and can produce a light beam that exhibits dynamic rotation around its center (azimuthal dimension) as well as in-and-out linear radial movement at a given propagation distance^27^; and (e) a light beam, which is created by driving the high-harmonic-generation of two time-delayed pulses carrying different OAM values, can exhibit dynamic rotation around its center at a time-dependent speed^29^. A laudable goal would be to produce a more sophisticated beam that can dynamically rotate and revolve at a tailorable speed and at a given propagation distance (Fig. 2).

**Fig. 3:**
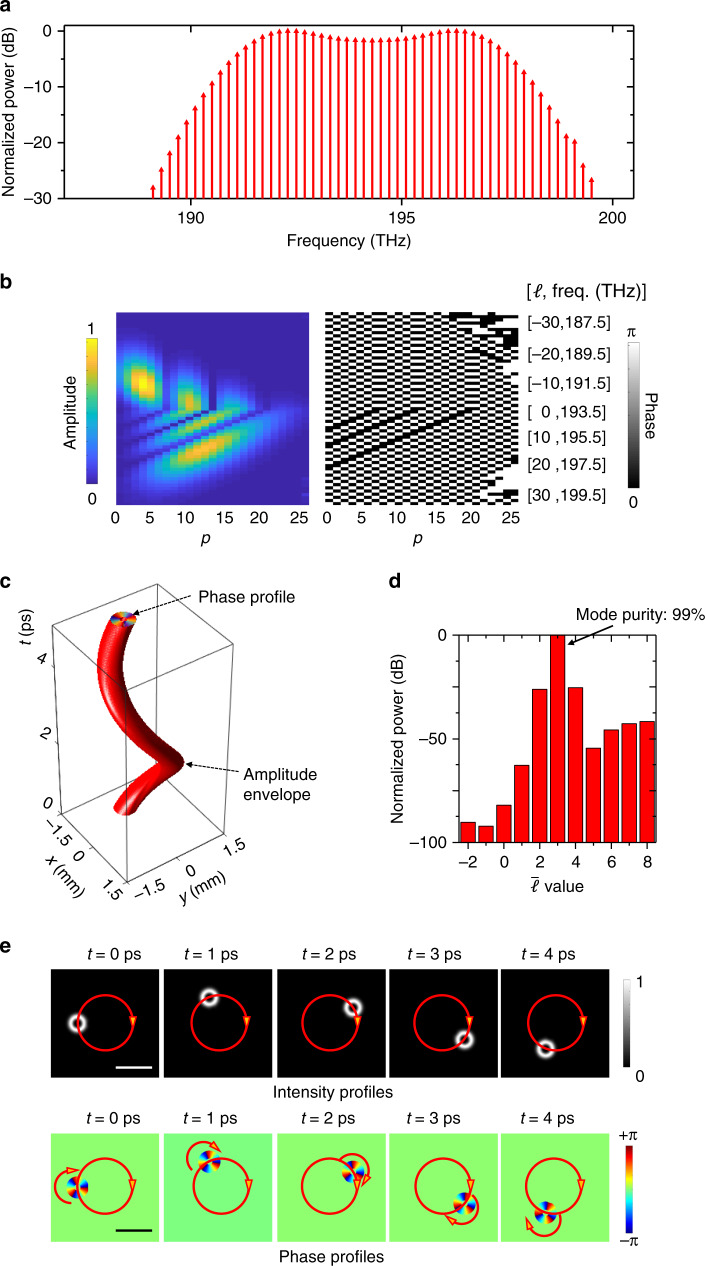
Simulation results of a spatiotemporal beam exhibiting both rotation and revolution at a given distance. For the generated rotating-revolving *LG*_3,0_ beam revolving at 0.2 THz, we simulate its **a** frequency spectrum; **b** spatial $LG_{\ell ,p}$ mode distribution, namely the amplitude and phase of the complex coefficients $C_{\ell ,p}$ of all the $LG_{\ell ,p}$ modes used for superposition; **c** envelope structure (i.e., the iso-surface with an amplitude of 1/10 of the peak value), where the top cap represents the helically twisting phasefront; **d** power distribution on light beams with different rotating $\bar \ell$ values; and **e** dynamically rotating and revolving intensity/phase profiles. Scale bar, 1 mm. The spatiotemporal beam consists of multiple frequency comb lines, in which each line is a superposition of multiple $LG_{\ell ,p}$ modes (same beam waist of 0.3 mm) with one $\ell$ value but multiple *p* values. The dynamic helical phasefront and envelope indicate that the beam not only rotates around its beam center but also revolves around another central axis 0.75 mm away from its center. freq.: frequency.

In this paper, we explore the generation of spatiotemporal light beams that combine two independent and controllable orbital angular momenta. This scenario is enabled by using multiple optical-frequency comb lines, with each line carrying a superposition of multiple $LG_{\ell ,p}$ modes containing a different $\ell$ value but multiple *p* values (Fig. 1f, j). As an example, we generate by simulation an $LG_{3,0}$ beam with a beam waist of *w*_0_ = 0.3 mm, which exhibits dynamic rotation around its beam center as well as revolution around a central axis with a revolving radius of *R* = 0.75 mm at a speed of *f*_*r*_ = 0.2 THz (Fig. 3). We show via simulation that we are able to control not only the spatiotemporal beam’s helically twisting phasefront but also its dynamic, two-dimensional (2D) motion of rotation and revolution at a given propagation distance. Specifically, we vary several parameters, including the rotating $\bar \ell$ value, revolving speed, revolving radius, and beam waist of the generated spatiotemporal light beams.

## Results

### Introducing dynamic rotation and revolution

There are different types of dynamic optical beams that can exhibit simultaneously two forms of orbital angular momenta. One example of such beam propagation is an $LG_{\bar \ell ,\bar p}$ beam rotating around its beam center while it also revolves around another central axis. We note that both such dynamic rotation and revolution can be described by the transverse OAM^11^; however, such transverse OAM can be decomposed into different OAM components, which are related to the rotation around beam’s center^15^ and the revolution around another central axis^24,25^, respectively. We refer these two momenta related to the two types of motions as two forms of orbital angular momenta in order to distinguish them.

Our goal below is to generate a self-rotating electric field at *z* = 0 (and more generally at a chosen distance), that also revolves around a central axis, **O**, distance *R* from its self-rotating axis at a speed of *f*_rev_ revolutions per second (or Hz) (i.e., the number of circles per second that the electric field revolves around **O**). Revolving the electric field at *z* = 0 of a conventional (i.e., self-rotating and not revolving) $LG_{\bar \ell ,\bar p}$ beam at a speed of *f*_rev_ with a revolving radius of *R*, we can obtain a rotating-revolving electric field (see Supplementary Note 6 for more details):

1 $$\begin{array}{c}E_1\left( {x,y,0,t} \right) = LG_{\bar \ell ,\bar p}^{{\mathrm{Cartesian}}}\left( {x\cos \varphi \left( t \right) - y\sin \varphi \left( t \right) + R,x\sin \varphi \left( t \right)} \right.\\ \left. { + \,y\cos \varphi \left( t \right),0;\omega _0,w_0} \right)\exp \left( {i\omega _0t} \right)\end{array}$$

We refer to the beam with such a dynamic electric field as a rotating-revolving $LG_{\bar \ell ,\bar p}$ beam (i.e., a beam that exhibits dynamic rotation around its beam center as well as revolution around a central axis). In this equation, *ω*_0_ = 2*πf*_0_ is the angular frequency, *w*_0_ is the beam waist, and $LG_{\bar \ell ,\bar p}^{{\mathrm{Cartesian}}}\left( {x,y,z;\omega _0,w_0} \right)$ is the electric field in Cartesian coordinates of a conventional $LG_{\bar \ell ,\bar p}$ beam. $\varphi \left( t \right) = \omega _{{\mathrm{rev}}}t = 2\pi f_{{\mathrm{rev}}}t$ represents the revolving angular speed, and ($x\cos \varphi \left( t \right) - y\sin \varphi \left( t \right)$ + $R,x\sin \varphi \left( t \right)$ + $y\cos \varphi \left( t \right),0;\omega _0,w_0$) is the coordinate transformation of (*x*, *y*, 0; *ω*_0_, *w*_0_) in a reference frame rotating in the transverse plane at *z* = 0.

The electric field at *z* = 0 of such a rotating-revolving $LG_{\bar \ell ,\bar p}$ beam can also be described as a superposition of multiple frequency comb lines with each line carrying a unique spatial pattern. It can be written in the form (see the “Methods” section and Supplementary Note 6 for more details):

2 $$\begin{array}{c}E_1\left( {x,y,0,t} \right) = {\sum} {\mathop {\sum}\limits_{\ell ,p} {C_{\ell ,p}} } LG_{\ell ,p}\left( {r,\theta ,0;\omega _0 + \ell \omega _{{\mathrm{rev}}},w_0} \right)\\ {\mathrm{exp}}\left( {i\left( {\omega _0 + \ell \omega _{{\mathrm{rev}}}} \right)t} \right)\end{array}$$

$LG_{\ell ,p}\left( {r,\theta ,0;\omega _0 + \ell \omega _{{\mathrm{rev}}},w_0} \right)$ is the electric field of an $LG_{\ell ,p}$ mode in cylindrical coordinates, where $r = \sqrt {x^2 + y^2}$ and *θ* = arctan(*y*/*x*), and (*x*, *y, z*, *t*) are the coordinate and time, respectively. For the $\ell$-th frequency line carrying an $LG_{\ell ,p}$ mode, $C_{\ell ,p}$ is the complex coefficient and $\omega _0 + \ell \omega _{{\mathrm{rev}}}$ is the angular frequency. Clearly, the expansion of Eq. (2) requires infinite number of modes to be a perfectly accurate one, but we will show below that a reasonable number of a few tens provides an acceptable accuracy, as testified by the purity of the rotating-revolving $LG_{\bar \ell ,\bar p}$ beam.

In general, any spatial beam can be generated by a superposition of multiple modes from a complete spatial modal basis set^36,37^, and the dynamic revolution and rotation motions in this paper can be realized by the judicial selection of spatial modes and frequencies with appropriate complex coefficients, as described in Eq. (2). As an illustrative example, we first consider the principle of generating a rotating-revolving $LG_{\bar \ell ,0}$ beam with a zero $\bar p$ value. The generation of an $LG_{\bar \ell ,0}$ beam that dynamically rotates and revolves in time at a given distance can be explained by the coherent interference among all the frequency comb lines that each line carries a superposition of multiple $LG_{\ell ,p}$ modes. The generation can be understood by considering that a simple $LG_{\bar \ell ,0}$ beam that rotates around its beam center should be firstly offset from the central axis and then be made to revolve around the original central axis, as the following two steps:

In the first step, we introduce the dynamic rotation with a spatial offset. A single frequency carrying an $LG_{\bar \ell ,0}$ mode can generate an $LG_{\bar \ell ,0}$ beam with a ring-like intensity profile and a twisting phasefront of ${\mathrm{exp}}(i\bar \ell \theta )$ in a circle around its beam center, which is located at the central axis (Fig. 2a). Such a phasefront leads to a Poynting vector with a non-zero azimuthal component. Because the Poynting vector indicates the propagation direction of light beams in free space, the phasefront of the above-generated beam dynamically rotates around its beam center, which is located at the central axis, in time at a given propagation distance^16^. Such a structured beam can keep its intensity and phase profiles, and be made offset by combining several modes from a complete $LG_{\ell ,p}$ modal basis set on a single frequency^38,39^. The approach is to choose an appropriate complex coefficient for each mode. As shown in Fig. 2b, the constructive interference of multiple $LG_{\ell ,p}$ modes on a single frequency produces a light beam with intensity and phase profiles as the same as those of an $LG_{\bar \ell ,0}$ beam, whose beam center is made radially offset from the central axis by a certain distance. Because the light beam still has a twisting phasefront of ${\mathrm{exp}}(i\bar \ell \theta )$, the phasefront dynamically rotates around its beam center, which is offset from the central axis, in time at a given distance.

In the second step, we introduce the dynamic revolution. For the above superposition, the relative phase delays among all the modes are time-invariant, thus the constructive interference produces an $LG_{\bar \ell ,0}$ beam whose intensity profiles appears static at any given point of propagation distance. An additional dynamic revolution around the central axis could be introduced by choosing appropriate time-variant relative phase delays among these modes. One possible approach is to combine different modes located on different frequencies. Here, we introduce a time-variant relative phase delay of Δ*φ* = 2πΔ*ft* between the neighboring $LG_{\ell ,p}$ modes (i.e., Δ$\ell$ = 1) by combining multiple frequency comb lines. Each frequency line carries multiple $LG_{\ell ,p}$ modes with a different $\ell$ value and multiple *p* values, where *f*_0_ is the center frequency, Δ*f* is the frequency spacing between neighboring frequency comb lines, and $\omega _\ell = 2\pi (f_0 + \ell \Delta f)$ is the angular frequency of each $LG_{\ell ,p}$ mode. In terms of the superposition of $LG_{\ell ,p}$ modes on a single frequency, previous work has found that introducing a relative phase delay of Δ*φ* between neighboring $LG_{\ell ,p}$ modes will rotate the azimuthal location of the generated light beam by an angle of Δ*θ* = Δ*φ*^38–40^. In our case, the time-variant relative phase delay will lead to dynamic constructive and destructive interferences, which produce an offset $LG_{\bar \ell ,0}$ beam exhibiting not only dynamic rotation around its beam center but also dynamic revolution around a central axis (see Fig. 2c).

### Generation of a rotating-revolving LG beam

Here, we detail the method for generating a rotating-revolving $LG_{\bar \ell ,0}$ beam. As an illustrative example, we simulate the dynamic motion of an *LG*_3,0_ beam (beam waist *w*_0_ = 0.3 mm, center frequency *f*_0_ = 193.5 THz) revolving around a central axis with a radius of *R* = 0.75 mm at a speed of *f*_rev_ = 0.2 THz. We use 61 frequency comb lines with a frequency spacing Δ*f* of 0.2 THz. Each line is a superposition of multiple $LG_{\ell ,p}$ modes containing one unique $\ell$ value and multiple *p* values, where *p* varies from 0 to 24. The electric field can be represented by $\mathop {\sum}\nolimits_{\ell = - 30}^{30} {\mathop {\sum}\nolimits_{p = 0}^{24} {C_{\ell ,p}} } LG_{\ell ,p}\left( {x,y,0,\omega _\ell } \right){\mathrm{exp}}(i\omega _\ell t)$ at distance *z* = 0, where $\omega _\ell = 2\pi (f_0 + \ell \Delta f)$ is linearly dependent on the azimuthal mode index $\ell$, and the frequency line at $\omega _\ell$ carries a superposition of spatial patterns $\mathop {\sum}\nolimits_{p = 0}^{24} {C_{\ell ,p}} LG_{\ell ,p}\left( {x,y,0,\omega _\ell } \right)$.

We characterize the beam’s spatial spectrum (i.e., spatial $LG_{\ell ,p}$ mode distribution) using the amplitude and phase of its complex coefficients $C_{\ell ,p}$ for each $LG_{\ell ,p}$ mode (Fig. 3b). Moreover, we map the spatial spectrum onto the frequency spectrum based on the linear relationship between the mode index $\ell$ and the angular frequency $\omega _\ell$ (Fig. 3a). Specifically, we calculate the total power on each frequency comb line using the total power of the superposition of $\mathop {\sum}\nolimits_{p = 0}^{24} {C_{\ell ,p}} LG_{\ell ,p}\left( {x,y,0,\omega _\ell } \right)$ (see Supplementary Fig. 1 for the spatial patterns on selected frequency lines). In addition, the phasefront and amplitude envelope (equi-amplitude surface) structures of such a beam are simulated (see Fig. 3c), in which the mode purity of the generated rotating-revolving *LG*_3,0_ beam is obtained to be ~99% (see Fig. 3d). As shown in Fig. 3e, the dynamic helical phasefront and amplitude profiles indicate that the beam exhibits both dynamic rotation and revolution in time at a given distance. (See Supplementary Video 1 for a real-time video of rotating-revolving $LG_{\bar \ell ,0}$ beams with different rotating $\bar \ell$ values.)

### Diffraction of a rotating-revolving LG beam

**Fig. 4:**
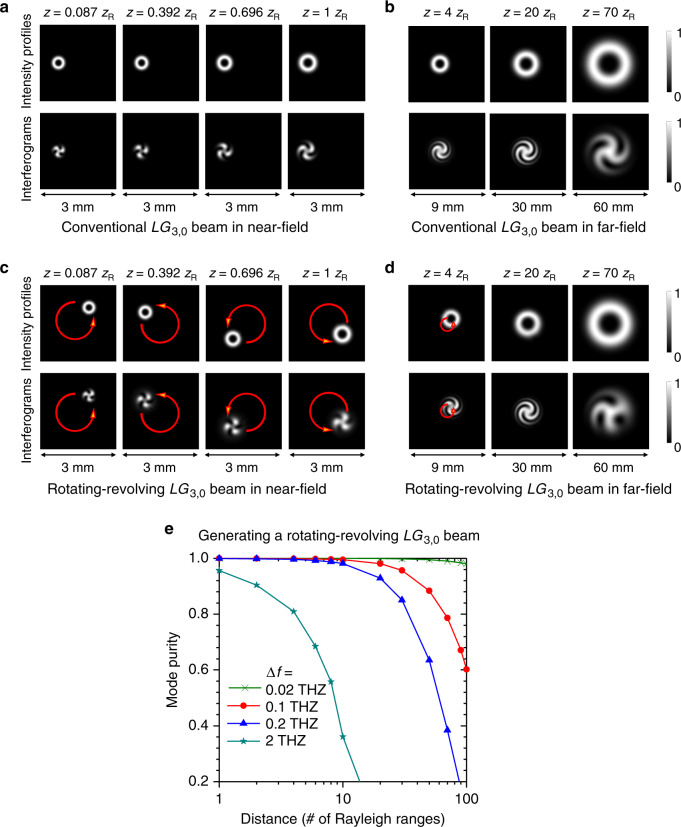
Simulation results of diffraction effects for a spatiotemporal beam exhibiting both rotation and revolution. Propagation over distance of an offset conventional *LG*_3,0_ in **a** the near-field and **b** the far-field for *t* = 0. Propagation along distance of a rotating-revolving *LG*_3,0_ in **c** the near-field and **d** the far-field for *t* = 0. The first and second rows in (**a**)–(**d**) are the intensity profiles of the propagating beams and the corresponding interferograms with Gaussian beams, respectively. As the propagation distance increases, the spatiotemporal beam counterclockwise revolves around the central axis. **e** The mode purity of a generated rotating-revolving *LG*_3,0_ beam with different revolving speed (0.02–2 THz) when the propagation distance is varied from 0 to 100*z*_R_, where *z*_R_ = 45.6 mm is the Rayleigh range.

To characterize the quality of the rotating-revolving *LG*_3,0_ beam at various propagation distances, we analyze the free-space diffraction effects in the near- and far-field. The examples in Fig. 4a–d show the comparison of the free-space propagation between an offset conventional $LG_{3,0}$ beam (beam center at (*x*, *y*) = (−0.75 mm, 0)) and the above-generated rotating-revolving *LG*_3,0_ beam (revolving speed *f*_rev_ = 0.2 THz, revolving radius *R* = 0.75 mm). The Rayleigh range is *z*_R_(*ω*_0_, *w*_0_) = 45.6 mm for the center frequency line. Figure 4c, d shows that the spatiotemporal beam counterclockwise revolves around the central axis as a function of *z* for *t* = 0 (see Supplementary Notes 5 and 7 for the analysis). Within the Rayleigh range, the shapes of the intensity profiles of the rotating-revolving *LG*_3,0_ beam and its interferograms with Gaussian beams are almost the same as those of a conventional *LG*_3,0_ beam. With further propagation, such as at a distance of 70*z*_R_, the interferogram of a conventional *LG*_3,0_ beam remains as a twisting shape, while the interferogram of the rotating-revolving *LG*_3,0_ beam is distorted. The mode purity of the rotating-revolving *LG*_3,0_ beam is >90% from 0 to 20*z*_R_; it decreases to 38% at 70*z*_R_ (see the blue curve in Fig. 4e). Figure 4e also shows that the propagation distance of the rotating-revolving *LG*_3,0_ beam with mode purity of >90% increases from 2*z*_R_ to more than 100*z*_R_, when the revolving speed *f*_*r*_ decreases from 2 to 0.02 THz.

The difference between the diffraction effects of an offset conventional *LG*_3,0_ beam and a rotating-revolving *LG*_3,0_ beam can be understood in the following manner:

- The electric field (*z* = 0) of an offset conventional *LG*_3,0_ beam and a rotating-revolving *LG*_3,0_ beam can be expressed as a superposition of multiple $LG_{\ell ,p}$ modes with the same mode distribution but different frequency spectra (i.e., one frequency line at *ω*_0_ or multiple frequency lines at $\omega _0 + \ell \omega _{{\mathrm{rev}}}$);
- When the frequency difference $\ell \omega _{{\mathrm{rev}}}$ is $\ll \omega _0$ and the beam is within the Rayleigh range, the diffraction effects of the $LG_{\ell ,p}$ mode carried by the frequency line at *ω*_0_ are almost the same as those of the same mode carried by the frequency line at $\omega _0 + \ell \omega _{{\mathrm{rev}}}$. Thus, the two superpositions with the same mode distribution but different frequency spectra are similar to each other in the near-field (see Supplementary Notes 5 and 7 for more analysis details); and
- However, such diffraction effects tend to differ with each other (i) with further propagation at far-field and (ii) as the frequency difference $\ell \omega _{{\mathrm{rev}}}$ increases. The diffraction difference might introduce different spatial amplitude and phase distortions to the same mode carried by the frequency lines on *ω*_0_ and $\omega _0 + \ell \omega _{{\mathrm{rev}}}$. As a result, the superposition of multiple modes carried by a single frequency line remains as an *LG*_3,0_ beam, while the superposition of multiple modes carried by multiple frequency lines is distorted; thus, the mode purity decreases.

### Control two orbital angular momenta

**Fig. 5:**
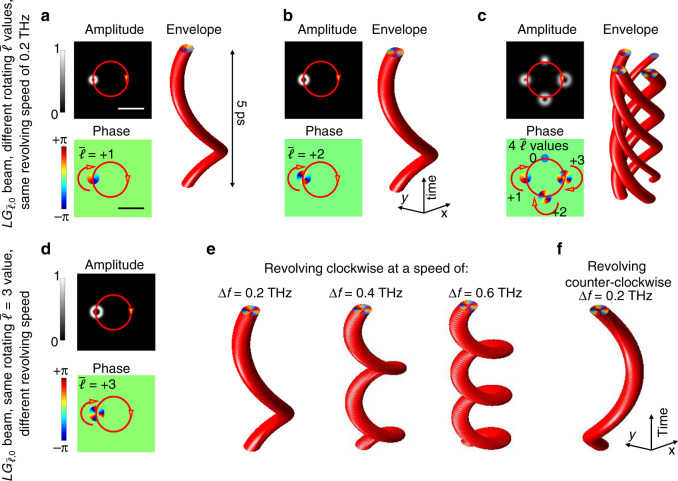
Independent control of two momenta of the spatiotemporal beams. **a**–**c** The 2D amplitude and phase profiles at time *t* = 0, and the envelope structures in (*x*, *y*, *t*) coordinates of the generated rotating-revolving $LG_{\bar \ell ,0}$ beams with different rotating $\bar \ell$ values but the same revolving speed of 0.2 THz. Scale bar, 1 mm. The phasefronts are exp(*iθ*) and exp(*i*2*θ*) in one circle around the beams’ center intensity nulls in (**a**) and (**b**), respectively; panel (**c**) is a single beam combining an array of four rotating-revolving $LG_{\bar \ell ,0}$ beams, where $\bar \ell = 0,1,2,3$. See Supplementary Fig. 2 for details of the spatial $LG_{\ell ,p}$ mode distributions used for superposition. **d** The corresponding profiles/structures of the generated rotating-revolving $LG_{\bar \ell ,0}$ beams with the same $\bar \ell$ = 3 value but a different revolving speed. **e** Examples of an *LG*_3,0_ beam revolving clockwise at different speeds from 0.2 to 0.6 THz; and **f** an *LG*_3,0_ beam revolving counterclockwise at a speed of 0.2 THz. Except for the varied parameters and the spatial/frequency spectra, all the other parameters are the same as those in Fig. 2.

Based on our simulations, the two momenta can be independently and separately controlled by tuning the rotating $\bar \ell$ values and the revolving speed of different rotating-revolving $LG_{\bar \ell ,0}$ beams. These two momenta are associated with the dynamic rotation and revolution, respectively (Fig. 5). Specifically, we investigate the cases for a rotating-revolving $LG_{\bar \ell ,0}$ beam (i) revolving clockwise at a speed of 0.2 THz and carrying a rotating $\bar \ell$ value varying from 1 to 3 (Fig. 5a–c), or (ii) carrying the same rotating $\bar \ell = 3$ value and revolving at a speed varying from 0.2 to 0.6 THz (Fig. 5d–f). Two phenomena can be discerned from Fig. 5. First, the rotating $\bar \ell$ value of the rotating-revolving $LG_{\bar \ell ,0}$ beam can be controlled by changing the spatial $LG_{\ell ,p}$ mode distribution carried by each frequency line (see Supplementary Fig. 2 for details). Second, the $LG_{\bar \ell ,0}$ beam revolves at a speed equal to the frequency spacing Δ*f*. This is because that the dynamic revolution is related to the time-variant relative phase delay between the neighboring $LG_{\ell ,p}$ mode for superposition, and its value is Δ*φ* = 2πΔ*ft*. Moreover, it is possible to change the direction of revolution of a rotating-revolving $LG_{\bar \ell ,0}$ beam by reassigning each modal combination $\mathop {\sum}\nolimits_p {C_{\ell ,p}} LG_{\ell ,p}\left( {x,y,0,\omega _\ell } \right)$, which is originally carried by a frequency line on $\omega _\ell = \omega _0 + \ell \omega _{{\mathrm{rev}}}$, to be carried by the one on $\omega _0 - \ell \omega _{{\mathrm{rev}}}$^12,13^. Therefore, the total amount of orbital angular momenta associated with these two motions could be independently controlled by changing the spatial $LG_{\ell ,p}$ mode distribution and frequency spectrum, respectively. (See Supplementary Fig. 3 for the cases of flipping the sign of the rotating $\bar \ell$ value and/or the revolving direction.)

**Fig. 6:**
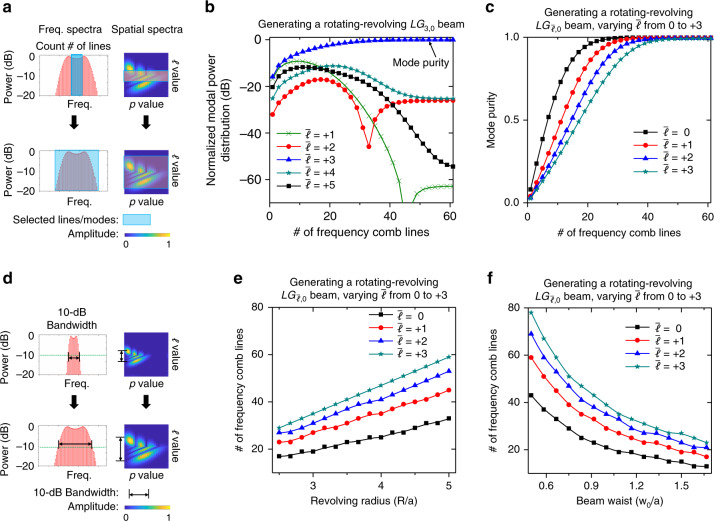
The relationship between the quality of the rotating-revolving $LG_{\bar {\ell ,0}}$ beams and the frequency spectrum. **a** We first calculate the spatiotemporal spectra of the rotating-revolving beam using the approach described in Methods, and then we select a certain number of frequency lines/modes from the calculated spectra for power/mode purity calculation in (**b**) and (**c**). **b** The power distribution on light beams with different rotating $\bar \ell$ values for generating a rotating-revolving *LG*_3,0_ beam, when the number of selected frequency comb lines is varied. **c** The mode purity of a generated rotating-revolving $LG_{\bar \ell ,0}$ beam ($\bar \ell$ varies from 0 to +3), when the number of selected frequency comb lines is varied. **d** We calculate the spatiotemporal spectra of different rotating-revolving beams and count the number of frequency lines in the 10-dB bandwidth of the frequency spectra; panels (**e**) and (**f**) show the number of frequency comb lines in the 10-dB bandwidth for generating a rotating-revolving $LG_{\bar \ell ,0}$ beam with (i) the same beam waist of *w*_0_ = 0.2 mm and a revolving radius of *R* varied from 0.75 to 1.5 mm; and (ii) the same revolving radius of *R* = 1.5 mm and a beam waist of *w*_0_ varied from 0.15 to 0.5 mm, respectively. a = 0.3 mm in (**e**) and (**f**).

Furthermore, we investigate the quality of the dynamic spatiotemporal beam with respect to the frequency spectrum. Here, all the frequency comb lines carry multiple $LG_{\ell ,p}$ modes with the same beam waist of 0.3 mm. Figure 6a–c shows the relationship between the power distribution on light beams with different rotating $\bar \ell$ values and the number of selected frequency comb lines. Figure 6b shows that when the number of comb lines is selected to be <10, the power coupling (the difference between the blue curve and other curves) to the light beams with the undesired rotating $\bar \ell \, \ne \, 3$ value is >−5 dB and the mode purity of the generated rotating-revolving *LG*_3,0_ beam is <25%; while when the number of comb lines is >40, the power coupling is <−20 dB and the mode purity is >95%. We can see from Fig. 6c that combining ~30 frequency lines could generate a rotating-revolving $LG_{\bar \ell ,0}$ beam, where $\bar \ell = 0,1,2,3$, with mode purity of >90%. For the cases where a limited number of frequency lines, such as 20, are used, the mode purity of the generated rotating-revolving $LG_{\bar \ell ,0}$ beam is higher for smaller rotating $\bar \ell$ values. Figure 6d–f shows the number of frequency comb lines within the 10-dB bandwidth of the frequency spectra for generating rotating-revolving $LG_{\bar \ell ,0}$ beams with different revolving radii or beam waists. The simulation results show that a larger number of frequency comb lines would generate a rotating-revolving $LG_{\bar \ell ,0}$ beam with a (i) larger revolving radius, (ii) smaller beam waist, or (iii) higher rotating $\bar \ell$ value. These relationships can be understood by referring to a Fourier transformation; by looking at the dynamic azimuthal mode (the generated spatiotemporal beam) at a given time, the beam can be described as a superposition of multiple $LG_{\ell ,p}$ modes with different azimuthal index $\ell$ values^38^. As the light beam’s (i) revolving radius increases, (ii) beam waist decreases, or (iii) rotating $\bar \ell$ value increases, the azimuthal mode will be spatially distributed within a smaller azimuthal range; thus the number of comb lines increases after applying a Fourier transformation from the azimuthal spatial domain to the frequency domain^38^.

## Discussion

We have explored the generation of a spatiotemporal light beam containing two independent orbital angular momenta using multiple frequency comb lines. Although our examples only focus on the generation of rotating-revolving $LG_{\bar \ell ,0}$ beams with a revolving speed of sub-THz, it might be possible to generate spatiotemporal light beams with different speeds and more sophisticated structures.

The speed of the dynamic motion could be controlled by tuning the frequency spacing between the frequency lines. It is thus possible to vary the revolving speed from several MHz to sub-THz by changing the frequency spacing of the frequency comb. Besides, if frequency lines with non-constant frequency spacing are coherently combined, the generated light beam might exhibit dynamic motions with time-variant speed.

The structure of the generated spatiotemporal light beam could be tuned by changing the spatial $LG_{\ell ,p}$ mode distribution. For example, it is possible to extend our method to generate rotating-revolving $LG_{\bar \ell ,\bar p}$ beams with non-zero $\bar p$ values. Moreover, if each frequency comb line carries a superposition of multiple $LG_{\ell ,p}$ modes containing both multiple $\ell$ values and multiple *p* values, it might be possible to simultaneously generate multiple rotating-revolving $LG_{\bar \ell ,\bar p}$ beams with different parameters, such as different non-zero $\bar p$ values, or revolving radii. In addition, a spatiotemporal light beam would experience spatial beam diffraction when propagating in free space. As a result, it might not maintain the same dynamic properties at different distances. It might be possible to generate a non-diffraction rotating-revolving Bessel beam through combining multiple frequency comb lines with each carrying multiple modes in the Bessel modal basis^41^.

We note that our analysis does not include the beam polarization since we are trying to isolate the effects of orbital angular momenta without considering spin angular momentum. However, we believe that it might be possible to generate a rotating-revolving $LG_{\bar \ell ,\bar p}$ beam that also carries spin angular momentum. One potential method could be realized in three steps: (i) generating two rotating-revolving $LG_{\bar \ell ,\bar p}$ beams on *x*- and *y*-polarizations, (ii) subsequently adding a phase delay of *π*/2 to one of the beams^24^, and (iii) finally coherently combining the two beams.

We also note that our results indicate that we might need to combine a large number of frequency comb lines with each carrying a large number of $LG_{\ell ,p}$ modes in order to generate a rotating-revolving $LG_{\bar \ell ,\bar p}$ beam with high mode purity. Although these large numbers are difficult to achieve at present, there have been reports of generating such large numbers of modes and frequency lines that could potentially be used for spatiotemporal light shaping. For example, reports have shown the generation and combination of (i) ~210 $LG_{\ell ,p}$ modes^42^, and (ii) ~90 frequency lines with each carrying different modes^43^. We believe those techniques indicate the potential to handle the experimental feasibility of the rotating-revolving $LG_{\ell ,p}$ beams with high mode purity.

## Methods

### Simulation details

The scalar electric field of an $LG_{\ell ,p}$ mode in cylindrical coordinates can be described by^16^:

3 $$LG_{\ell ,p}\left( {r,\theta ,z;\omega ,w_0} \right) 	= U\left( {r,z;\omega ,w_0} \right){\mathrm{exp}}(i\ell \theta )\\ 	= \frac{{C_{\ell ,p}^{{\mathrm{LG}}}}}{{w\left( {z,\omega } \right)}}\left( {\frac{{r\sqrt 2 }}{{w\left( {z,\omega } \right)}}} \right)^{\left| \ell \right|}\exp \left( { - \frac{{r^2}}{{w^2\left( {z,\omega } \right)}}} \right)L_p^{\left| \ell \right|}\left( {\frac{{2r^2}}{{w^2\left( {z,\omega } \right)}}} \right)\\ 	\quad {\mathrm{exp}}\left( { - i\left( {k\frac{{r^2}}{{2R\left( {z,\omega } \right)}} + kz - \ell \theta - \psi \left( {z,\omega } \right)} \right)} \right)$$

where *U*(*r*, *z*; *ω*, *w*_0_) is the complex electric field independent with *θ*, $L_p^{\left| \ell \right|}$ are the generalized Laguerre polynomials, and $C_{\ell ,p}^{{\mathrm{LG}}}$ are the required normalization constants, $w\left( {z,\omega } \right) = w_0\sqrt {1 + \left( {z/z_{\mathrm{{R}}}(\omega ,w_0)} \right)^2}$ is the beam waist, and $R\left( {z,\omega } \right) = z(1 + (z_{\mathrm{R}}(\omega ,w_0)/z)^2)$, where $z_{\mathrm{R}}\left( {\omega ,w_0} \right) = \omega w_0^2{\mathrm{/}}2c$ is the Rayleigh range in free space, *k* is the wave number; and *ψ*(*z*) is the Gouy phase and equals $\left( {\left| \ell \right| + 2p + 1} \right)\arctan \left( {z/z_{\mathrm{R}}(\omega )} \right)$. The parameters (*r*, *θ*, *z*; *ω*, *w*_0_) have the same definitions as in the “Results”.

We numerically generate the spatiotemporal beam in three steps: (i) we first calculate the complex spatial mode distribution of a conventional $LG_{\bar \ell ,0}$ beam centered at (*x*, *y*) = (−*R*, 0) by decomposing its electric field into an $LG_{\ell ,p}$ mode basis centered at (*x*, *y*) = (0, 0). The frequency is *f*_0_, the revolving radius is *R*, *z* = 0, and *t* = 0; (ii) we then coherently combine all the spatial modes with the same $\ell$ value but different *p* values to obtain the spatial pattern of the frequency line at $\omega _\ell = 2\pi (f_0 + \ell \Delta f)$, where Δ*f* is the revolving speed; (iii) we calculate the electric field of the rotating-revolving $LG_{\bar \ell ,0}$ beam by coherently combining the electric fields of all the frequency comb lines. We only consider the cases in which the frequency separation Δ*f* is a constant and the center frequency is 193.5 THz. In our simulation model, there are 500 × 500 pixels with a 6-μm pixel size in the (*x*, *y*) plane, and 400 pixels with a 12.5-fs pixel size in time.

### Mode purity calculation

Considering that the observed intensity and phase profiles of the generated rotating-revolving $LG_{\bar \ell ,0}$ beams remain relatively invariant if an observer moves dynamically with the rotating-revolving beams (Fig. 2f), we calculate the mode purity as the normalized power weight coefficient of the generated spatiotemporal beam at time *t* = 0 and distance *z* = 0 using $|C_\ell |^2 = \left| {{\iint} {E_1\left( {x,y} \right)} E_2^ \ast \left( {x,y} \right)dxdy} \right|^2$^9^, where *E*_1_(*x*, *y*) is the generated electric field of the generated spatiotemporal beam, and *E*_2_(*x*, *y*) is the electric field of a conventional $LG_{\ell ,0}$ beam with center overlapping with the generated beam, the operator * denotes the conjugation calculation. Both *E*_1_(*x*, *y*) and *E*_2_(*x*, *y*) are normalized, namely, $\left| { {\iint}{E_i\left( {x,y} \right)} E_i^ \ast \left( {x,y} \right)dxdy} \right|^2 = 1$, where *i* = 1 or 2. We calculate the $|C_\ell |^2$ using the integral, (i) over the whole transverse plane when the beam is within the Rayleigh range, and (ii) over a ring-shape area with a radius from 0.9*R*_max_ to 1.1*R*_max_ (where *R*_max_ is the distance from the intensity peak to the beam center) outside the Rayleigh range. Here, the calculated mode purity represents the ratio between the power on the spatiotemporal beam with the desired rotating $\bar \ell$ value and the total power of the generated beam.

### Generalization for the generation of rotating-revolving LG beams

We have shown in the Results some special cases as illustrative examples of the generation of rotating-revolving $LG_{\bar \ell ,\bar p}$ beams. However, it is interesting to consider the generalization of our generation method to a broader range so that it can generate a rotating-revolving $LG_{\bar \ell ,\bar p}$ beams with other $(\bar \ell ,\bar p)$ values (e.g., $\bar \ell$ values of >10 or non-zero $\bar p$ values). A rotating-revolving $LG_{\bar \ell ,\bar p}$ beam can be generated by offsetting a conventional $LG_{\bar \ell ,\bar p}$ beam to have an electric field of $\psi \left( {x,y,0} \right){\mathrm{exp}}\left( {i\omega _0t} \right)$ at *z* = 0 and subsequently dynamically revolving the beam around a central axis. According to the modal decomposition method^36,37^, any offset conventional $LG_{\bar \ell ,\bar p}$ beam with arbitrary $(\bar \ell ,\bar p)$ values can be represented by a combination of multiple $LG_{\ell ,p}$ modes, namely, $E_0\left( {x,y,0,t} \right)$ = $\psi \left( {x,y,0} \right){\mathrm{exp}}\left( {i\omega _0t} \right)$ = ${\sum} {\mathop {\sum}\nolimits_{\ell ,p} {C_{\ell ,p}} } LG_{\ell ,p}\left( {r,\theta ,0;\omega _0,w_0} \right){\mathrm{exp}}\left( {i\omega _0t} \right)$. When the beam revolves clockwise around the origin at a speed of *f*_*r*_ revolutions per second, the revolution motion introduces a frequency shift of $\ell \omega _r$ to each $LG_{\ell ,p}$ mode so that *ω*_0_ shifts to $\omega _0 + \ell \omega _{{\mathrm{rev}}}$^13,44^. Such a frequency shift transforms the electric field of a single frequency line carrying multiple modes into the form

4 $$E_1\left( {x,y,0,t} \right) = {\sum} {\mathop {\sum}\limits_{\ell ,p} {} C_{\ell ,p}} LG_{\ell ,p}\left( {r,\theta ,0;\omega _0 + \ell \omega _{{\mathrm{rev}}},w_0} \right){\mathrm{exp}}\left( {i\left( {\omega _0 + \ell \omega _{{\mathrm{rev}}}} \right)t} \right)$$

which is the electric field at *z* = 0 of a rotating-revolving $LG_{\bar \ell ,\bar p}$ beam with arbitrary $(\bar \ell ,\bar p)$ values (see Supplementary Note 6 for details). Equation (4) indicates that a rotating-revolving $LG_{\bar \ell ,\bar p}$ beam can be generated by combining multiple frequency comb lines with each carrying multiple $LG_{\ell ,p}$ modes. This method could be generalized to generate rotating-revolving $LG_{\bar \ell ,\bar p}$ beams with any $(\bar \ell ,\bar p)$ values, by judiciously selecting the coefficient $C_{\ell ,p}$ to be an integral ${\iint} \psi \left( {x,y,0} \right)( {LG_{\ell ,p}\left( {r,\theta ,0;\omega _0,w_0} \right)} )^ \ast dxdy$^9,36,37^. However, when the $(\bar \ell ,\bar p)$ values increase, the coefficient $C_{\ell ,p}$ might still have nonnegligible values for $LG_{\ell ,p}$ modes with modal indices out of the ranges shown in the Article. Thus, in this case, we believe that a higher number of $LG_{\ell ,p}$ modes should be utilized for the combination to generate a rotating-revolving $LG_{\bar \ell ,\bar p}$ beam.

## Supplementary information

Supplementary Information

Description of Additional Supplementary Files

Supplementary Movie 1

## Data Availability

All data, theory details, simulation details that support the findings of this study are available from the corresponding authors on reasonable request.
