# Supplementary material for: Dynamic spatiotemporal beams that combine two independent and controllable orbital-angular-momenta using multiple optical-frequency-comb lines
Source: Nat Commun. 2020 Aug 14;11:4099. doi: 10.1038/s41467-020-17805-1 (PMC7427811; doi:10.1038/s41467-020-17805-1)
Supplement: Supplementary file 1 — Supplementary Information [file 41467_2020_17805_MOESM1_ESM.pdf]

Supplementary Information: Dynamic Spatiotemporal Beams that Combine Two Independent and Controllable Orbital-Angular-Momenta Using Multiple Optical-Frequency-Comb Lines

Zhe Zhao[1,*], Hao Song[1], Runzhou Zhang[1], Kai Pang[1], Cong Liu[1], Haoqian Song[1], Ahmed Almaiman[1,2], Karapet Manukyan[1], Huibin Zhou[1], Brittany Lynn[3], Robert W. Boyd[4,5], Moshe Tur[6], and Alan E. Willner[1,*]

1. *Department of Electrical Engineering, Univ. of Southern California, Los Angeles, CA 90089, USA*
2. *King Saud University, Riyadh 11362, Saudi Arabia*
3. *Naval Information Warfare Center Pacific, San Diego, CA, 92152, USA*
4. *Department of Physics, University of Ottawa, Ottawa, ON, Canada*
5. *The Institute of Optics, University of Rochester, Rochester, New York 14627, USA*
6. *School of Electrical Engineering, Tel Aviv University, Ramat Aviv 69978, Israel*

Corresponding emails: zhezhao@usc.edu, willner@usc.edu

Supplementary Note 1. Introduction

In this supplementary, we investigate more properties of the generated rotating-revolving $LG_{\bar{\ell},0}$ beam that combines two orbital-angular-momenta. Except for the results shown in the Article, we show: (i) the electric fields of the selected frequency comb lines for generating a rotating-revolving $LG_{3,0}$ beam; (ii) the approaches to independently control two orbital-angular-momenta of a generated rotating-revolving $LG_{\bar{\ell},0}$ beams; and (iii) the mode purity of a generated rotating-revolving $LG_{3,0}$ beam with respect to the frequency spectrum shape. These results show that: (a) each frequency comb line at f carries multiple $LG_{\ell,p}$ modes with a different ℓ value, which is proportional to f; (b) independent control of two orbital-angular-momenta can be achieved by appropriate choosing the spatial $LG_{\ell,p}$ mode distribution and the frequency spacing.

Supplementary Note 2. The generation of the rotating-revolving $LG_{3,0}$ beam

Except for the spatial $LG_{\ell,p}$ mode distribution as shown in Fig. 3 in the Article, we also simulate the spatial electric fields of the used frequency comb lines for generating a rotating-revolving $LG_{3,0}$ beam. As shown in Supplementary Figure 1, we calculate the electric fields of the selected frequency lines at distance $z = 0$ and time $t = 0$ by using $\sum_{p=0}^{25} C_{\ell,p} LG_{\ell,p}(x, y, 0, \omega_\ell)$, which is modified from Eq. (2) for generating the spatiotemporal beam in Fig. 2 in the Article. As expected, each frequency line has a unique "twisting" phase profile. The azimuthal ℓ values carried by the spatial patterns located on the 10th to 45th frequency lines are -20, -15, -10, -5, 0, +5, +10, and +15, respectively. These results clearly indicate that each frequency line has a helical phasefront of $\exp(i\bar{\ell}\theta)$, whose ℓ value is linearly dependent on the frequency. It can be noticed that some frequency comb lines have a multiple-concentric-ring intensity profile, and that the phase changes by π between neighboring rings along the radial direction. This can be explained by: (i) all the modes have the same helical phasefront of $\exp(i\bar{\ell}\theta)$ along the azimuthal direction, thus the interference of these modes still remains the same phasefront along the azimuthal direction; (ii) each mode carrying a different p value has different radial amplitude and phase profiles along the radial direction, thus the interference further results in a unique radial intensity and phase distribution along the radial direction.

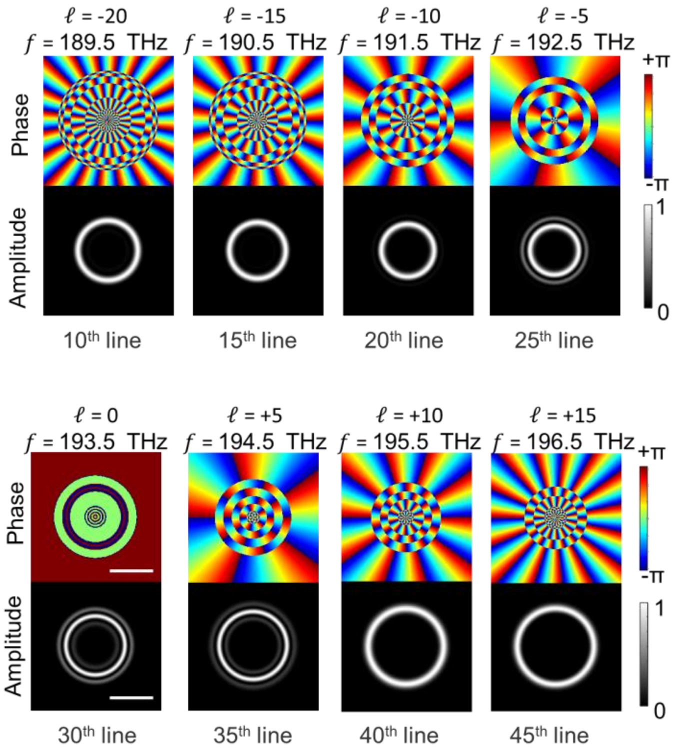

Supplementary Fig. 1 The spatial patterns on the selected frequency lines. The patterns for generating the rotating-revolving $LG_{3,0}$ beam in Fig. 2 in the Article. Each frequency comb line carries a superposition of multiple $LG_{\ell,p}$ modes with a different ℓ value and multiple p values.

Supplementary Note 3. Control the two orbital-angular-momenta

We also show more details about the approaches to independently control the two momenta of a rotating-revolving $LG_{\bar{\ell},0}$ beam, which are associated with the dynamic rotation and revolution, respectively. We show the simulated phasefronts and amplitude envelopes of the generated spatiotemporal light beams with different values of the two orbital-angular-momenta, as shown in Fig. 3 in the Article. We describe the approaches to achieve such independent control in the following paragraphs.

We first simulate the spatial $LG_{\ell,p}$ mode distributions for generating rotating-revolving $LG_{1,0}$ and $LG_{2,0}$ beams and an array of four rotating-revolving $LG_{\bar{\ell},0}$ beams with a revolving radius of 0.75 mm. The corresponding phase and intensity profiles of these beams are shown in Fig. 5 in the Article. As shown in Supplementary Figure 2, for the case of generating a rotating-revolving $LG_{\ell,0}$ beam with a non-zero rotating

$\bar{\ell}$ value, the spatial $LG_{\ell,p}$ mode distribution resembles a "heart" shape, which is then split into a number of $\bar{\ell}$ main parts. The reason that why the "heart" shape is split into $\bar{\ell}$ main parts might be explained by: the helical phasefront of $\exp(i\bar{\ell}\theta)$ has a periodic phase change ($\bar{\ell}$ periods) along the azimuthal direction; transforming such a periodic spatial shape into the $LG_{\ell,p}$ mode distribution is similar to a Fourier transformation, which splits the shape into $\bar{\ell}$ main parts. As for the case of generating an array of rotating-revolving $LG_{\bar{\ell},0}$ beams with multiple $\bar{\ell}$ values, there are several horizontal "noisy" lines in the "heart" shape, and most of its power is limited in a smaller number of $LG_{\ell,p}$ modes (Supplementary Figure 2c). Moreover, the generation method can be also extended to generate rotating-revolving $LG_{\bar{\ell},\bar{p}}$ beams non-zero $\bar{p}$ values and $\bar{\ell}$ values of >10. As examples, we simulate spatial $LG_{\ell,p}$ mode distributions for generating rotating-revolving $LG_{3,5}$ and $LG_{80,0}$ beams with a beam waist of 0.3 mm and a revolving radius of 0.5 mm (Supplementary Figure 2d, 2e). To generate the rotating-revolving $LG_{3,5}$ beam, we combine < 60 frequency lines, with each carrying multiple $LG_{\ell,p}$ modes with one ℓ value (-30 to +30) and multiple p values (0 to 24); For generating the rotating-revolving $LG_{80,0}$ beam, we combine ~90 frequency lines with each carrying multiple $LG_{\ell,p}$ modes with one ℓ value (-15 to +75) and multiple p values (0 to 9). These results show that in order to change the rotating $\bar{\ell}$ value of the generated rotating-revolving $LG_{\ell,p}$ beam, we need to change both the amplitude and phase of the complex coefficients $C_{\ell,p}$ of the spatial $LG_{\ell,p}$ mode distribution used for superposition.

We also show the dependence of the revolving speed of the generated rotating-revolving $LG_{3,0}$ beam on the frequency spacing Δf, as shown in Supplementary Figure 3a. The corresponding amplitude envelope is shown in Fig. 4 in the Article. The revolving speed is tuned from 0.2 THz to 0.7 THz, which is equal to the frequency spacing Δf. This is because the dynamic revolution is introduced by the time-variant relative phase delay between neighbouring $LG_{\ell,p}$ modes (*i.e.*, $\Delta\ell = 1$). The relative phase delay is equal to $2\pi\Delta f t$ such that the constructive interference of all the modes and frequency comb lines results in an intensity profiles revolving at a speed of Δf. We can see from Supplementary Figure 2that the frequency spacing need to be controlled in

order to tune the revolution speed of a rotating-revolving $LG_{\bar{\ell},0}$ beam. Additionally, we can independently change the sign of the values for two momenta by two steps: (i) changing the modal combination $\sum_p C_{\ell,p} LG_{\ell,p}(x, y, 0, \omega_\ell)$ carried by the frequency line on $\omega_\ell = \omega_0 + \ell\omega_r$ to another modal combination $\sum_p C_{\ell,p} LG_{-\ell,p}(x, y, 0, \omega_\ell)$ (*i.e.*, flipping the sign of ℓ value for each $LG_{\ell,p}$ mode); and/or (ii) reassigning each modal combination $\sum_p C_{\ell,p} LG_{\ell,p}(x, y, 0, \omega_\ell)$, which is originally carried by a frequency line on $\omega_\ell = \omega_0 + \ell\omega_{\mathrm{rev}}$, to be carried by the one on $\omega_0 - \ell\omega_{\mathrm{rev}}$. As compared to the clockwise-revolving $LG_{3,0}$ beam in Supplementary Figure 3b, we flip: (a) only the sign of the rotating ℓ value using step (i) in Supplementary Figure 3c; (b) only the revolving direction using step (ii) in Supplementary Figure 3d; and (iii) both the sign of the rotating ℓ value and revolving direction using steps (i) and (ii) in Supplementary Figure 3e.

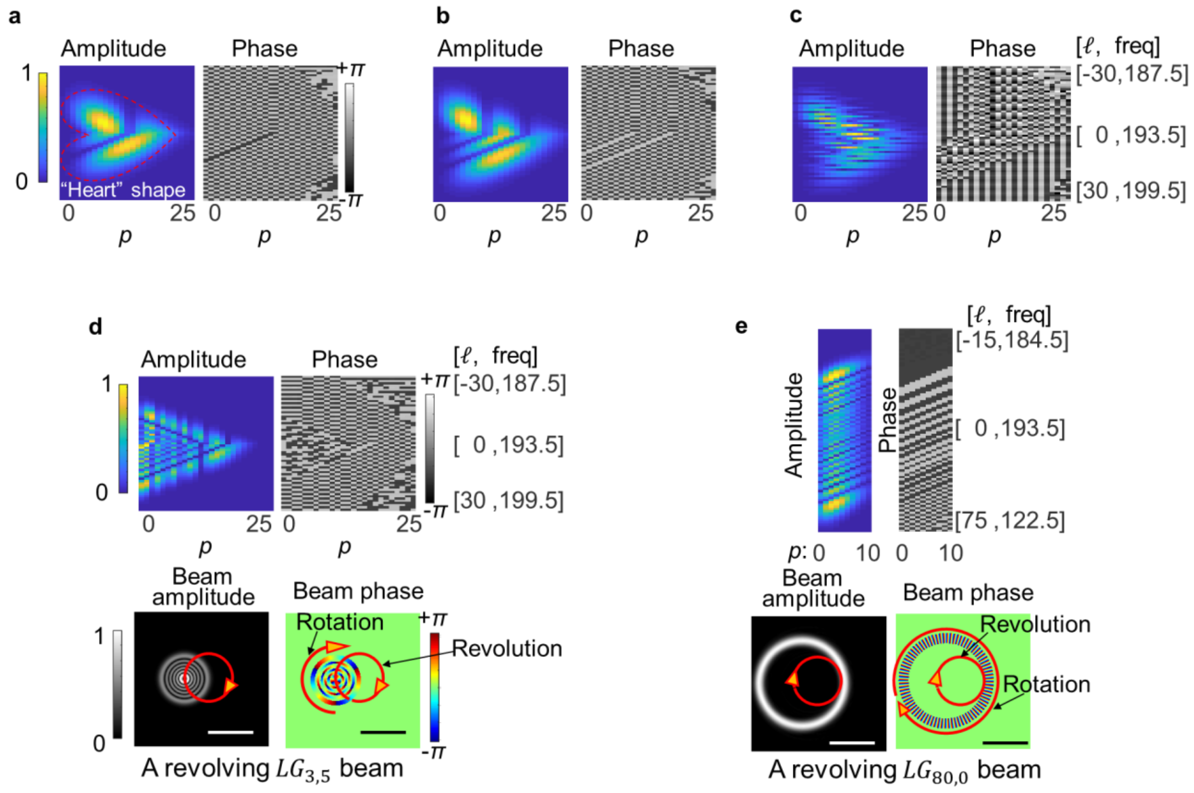

Supplementary Fig. 2 Simulated results for controlling the $\bar{\ell}$ and $\bar{p}$ values for rotating-revolving $LG_{\bar{\ell},\bar{p}}$ beams. (**a-c**) The spatial $LG_{\ell,p}$ mode distributions, namely the amplitude and phase of the complex coefficients $C_{\ell,p}$ of all the $LG_{\ell,p}$ modes, for generating the three different rotating-revolving light beams

shown in Fig. 5c in the Article. For generating rotating-revolving (**a**) $LG_{1,0}$ and (**b**) $LG_{2,0}$ beams, the amplitude matrices of the spatial $LG_{\ell,p}$ mode distributions are split into two and three main parts, respectively. (**c**) The spatial $LG_{\ell,p}$ mode distribution for generating an array of four rotating-revolving $LG_{\bar{\ell},\bar{p}}$ beams with $\bar{\ell} = 0, 1, 2, \text{and } 3$. (**d**) and (**e**) The spatial $LG_{\ell,p}$ mode distribution for generating a rotating-revolving $LG_{3,5}$ beam and $LG_{80,0}$ beam, respectively. Scale bar, 1 mm. freq: frequency.

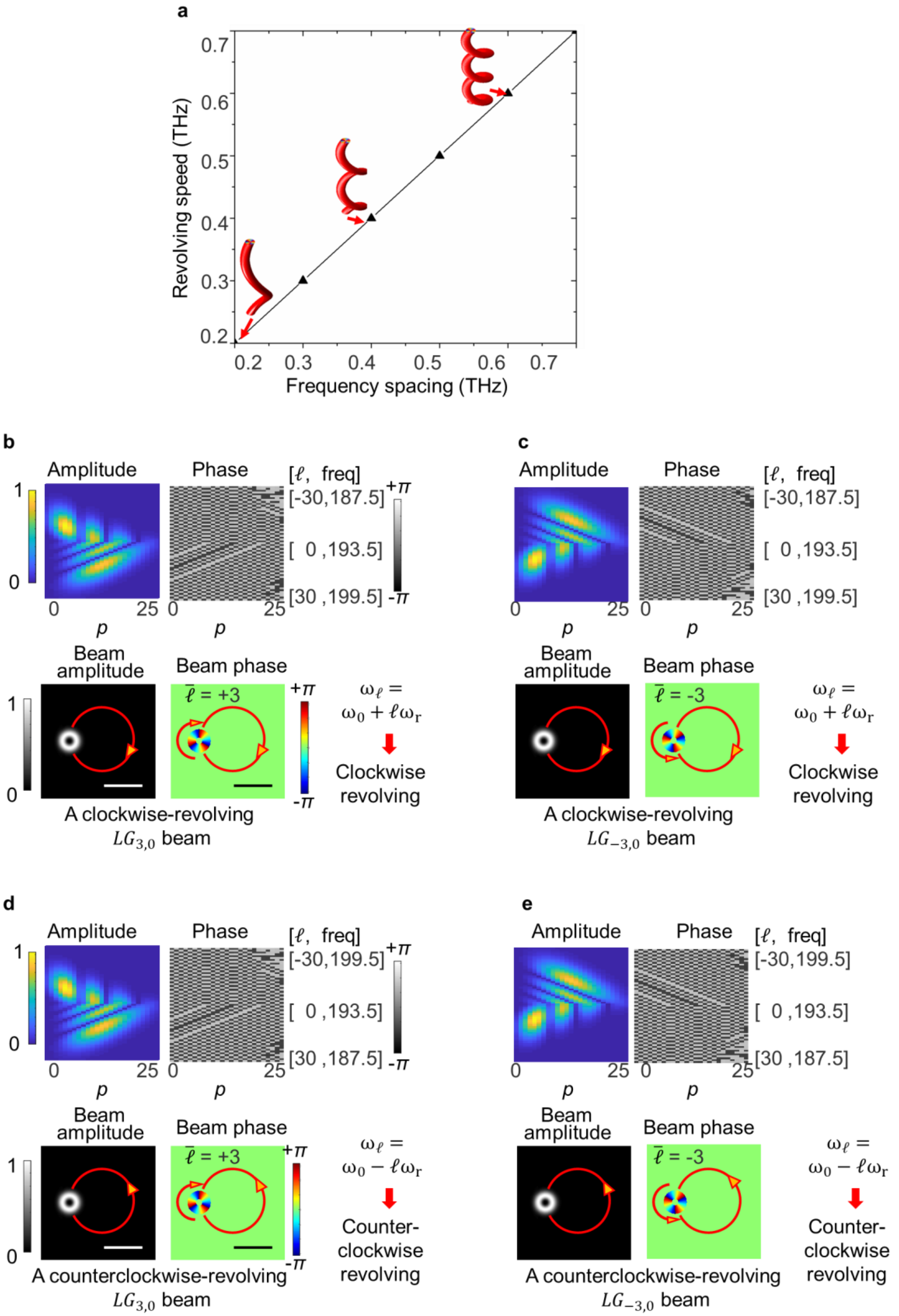

Supplementary Fig. 3 Simulated results for controlling the revolving speed and direction for rotating-revolving $LG_{\bar{\ell},\bar{p}}$ beams. (**a**) The revolving speed of such a rotating-revolving $LG_{3,0}$ beam is equal to the

frequency spacing. (**b-e**) The spatial $LG_{\ell,p}$ mode distributions, namely the amplitude and phase of the complex coefficients $C_{\ell,p}$ of all the $LG_{\ell,p}$ modes, for generating (**b**) clockwise-revolving $LG_{3,0}$, (**c**) clockwise-revolving $LG_{-3,0}$, (**d**) counterclockwise-revolving $LG_{3,0}$, (**e**) counterclockwise -revolving $LG_{-3,0}$. Scale bar, 1 mm. freq: frequency.

Supplementary Note 4. Mode purity of the generated spatiotemporal light beams

We also characterize the quality of the generated beams by analyzing the mode purity respect to different frequency spectrum shapes (Supplementary Figure 4). We focus on the quality of the dynamic spatiotemporal beam with respect to the number of frequency comb lines, as shown in Fig. 3 in the Article. One more interesting issue is the influence of the frequency spectrum shape on the mode purity. We still calculate the mode purity as the normalized power weight coefficients of the generated vortices at distance $z = 0$ and time $t = 0$. Each comb line carries the same spatial pattern, as shown in Fig. 3b in the Article (see selected patterns in Supplementary Figure 1), but the total power of each frequency line is varied. Supplementary Figure 4shows that changing the frequency spectrum shape will affect the mode purity if the generated spatiotemporal beam. The mode purity of the beam with the shape shown in Supplementary Figure 4dis ~ 96%. Such a shape is calculated based on the correlation between its spatial and frequency spectra, namely, that the frequency line at ω_ℓ carries a superposition of spatial patterns $\sum_{p=0}^{24} C_{\ell,p} LG_{\ell,p}(x, y, 0, \omega_\ell)$. However, the mode purity decreases to ~ 37%, 38%, and 3% for the shapes in Supplementary Figure 4a-c, respectively. The third shape has the lowest mode purity because it filters most of the power of the $LG_{\ell,p}$ modes, leading to higher spatial phase distortion. Supplementary Figure 4shows that in order to generate a rotating-revolving $LG_{\bar{\ell},0}$ beam with high mode purity (> 90%), the frequency spectrum needs to be shaped based on the correlation between its spatial and frequency spectra.

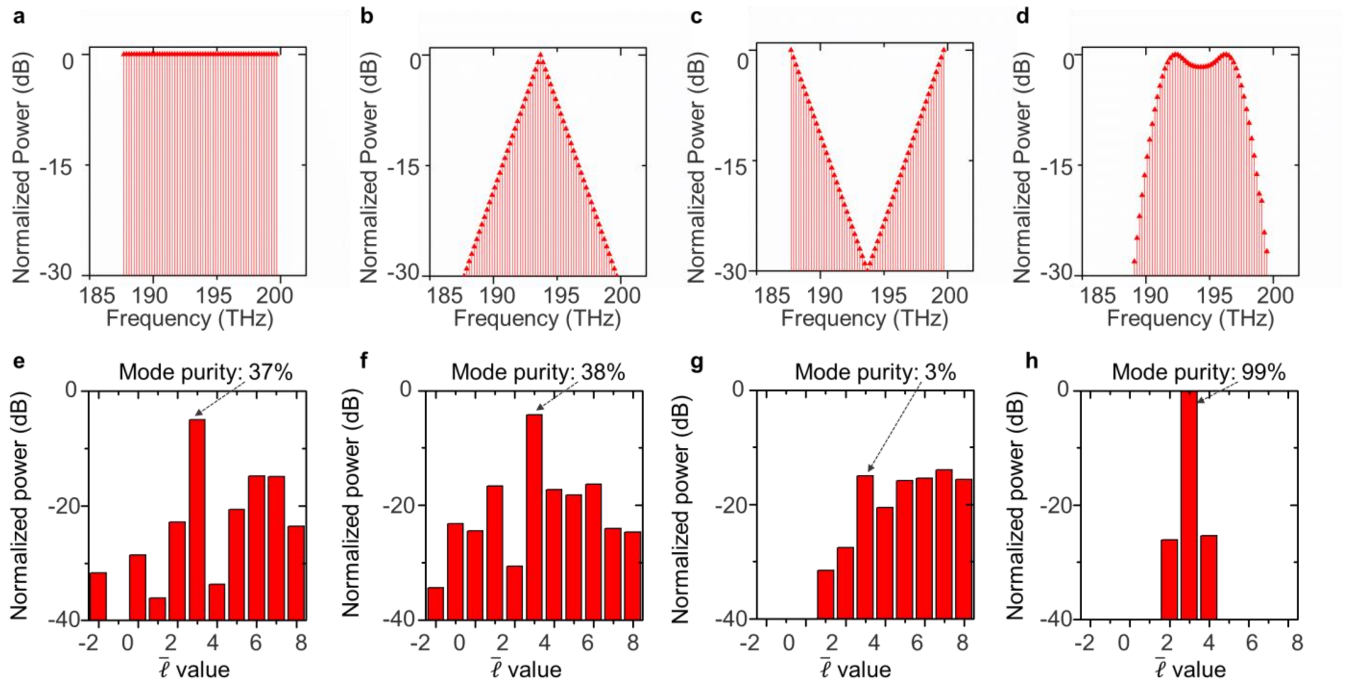

Supplementary Fig. 4 Effect of the frequency spectrum shaping on the normalized power distributions of light beams with different rotating $\bar{\ell}$ values. (**a**)-(**d**) and (**e**)-(**h**) are the frequency spectra with different shapes and the corresponding calculated normalized power distributions, respectively. These frequency comb lines are used to generate a rotating-revolving $LG_{3,0}$ beam. (**d**) is the same one shown in Fig. 3 in the Article.

Supplementary Note 5. Approximation of propagation for a single LG mode with small frequency shift

Here, we show the calculation details for estimating the electric field of a single free-space propagating $LG_{\ell,p}$ mode with a small frequency shift. Specifically, when $|l\omega_{\text{rev}}| \ll \omega_0$, $LG_{\ell,p}(r,\theta,z;\omega_0+\ell\omega_{\text{rev}},w_0) \approx LG_{\ell,p}(r,\theta,z;\omega_0,w_0)\exp\left(-i\,\ell\omega_{\text{rev}}z/c\right)$ at least in the Rayleigh range within the paraxial approximation, where the parameters z and r have limited values. Such approximation may not hold when the values of z and r reach infinity. The approximation in the Rayleigh range within the paraxial approximation is calculated as the expression of

$$\lim_{\frac{\ell\omega_{\text{rev}}}{\omega_0}\to 0} LG_{\ell,p}(r,\theta,z;\omega_0+\ell\omega_{\text{rev}},w_0) = \lim_{\frac{\ell\omega_{\text{rev}}}{\omega_0}\to 0} \left|LG_{\ell,p}(r,\theta,z;\omega_0+\ell\omega_{\text{rev}},w_0)\right|$$

$$\times \exp\left(i\angle\left(LG_{\ell,p}(r,\theta,z;\omega_0+\ell\omega_{\text{rev}},w_0)\right)\right) \quad (1)$$

where applying the angle operator ∠ leads to the spatial phase of an $LG_{\ell,p}$ mode.

We separately calculate the approximation for the amplitude part $\left|LG_{\ell,p}(r,\theta,z;\omega_0+\ell\omega_{\text{rev}},w_0)\right|$ and the phase part $\angle\left(LG_{\ell,p}(r,\theta,z;\omega_0+\ell\omega_{\text{rev}},w_0)\right)$:

(i) Limit calculation for the amplitude profile of the electric field.

We first consider the limit of the ratio of the beam waist parameters $w(z,\omega_0+\ell\omega_{\text{rev}})/w(z,\omega_0)$, which is

$$\lim_{\frac{\ell\omega_{\text{rev}}}{\omega_0}\to 0}\frac{w(z,\omega_0+\ell\omega_{\text{rev}})}{w(z,\omega_0)}=\lim_{\frac{\ell\omega_{\text{rev}}}{\omega_0}\to 0}\sqrt{\frac{1+\left(\frac{z}{z_{\text{R}}(\omega_0+\ell\omega_{\text{rev}},w_0)}\right)^2}{1+\left(\frac{z}{z_{\text{R}}(\omega_0,w_0)}\right)^2}}=$$

$$\lim_{\ell\omega_{\text{rev}}/\omega_0\to 0}\sqrt{1+\frac{\left(\frac{z}{w_0{}^2/2c}\right)^2\left(\frac{1}{\omega_0+l\omega_{\text{rev}}}+\frac{1}{\omega_0}\right)\frac{1}{(\omega_0+\ell\omega_{\text{rev}})}\frac{-\ell\omega_{\text{rev}}}{\omega_0}}{1+\left(\frac{z}{\omega_0 w_0{}^2/2c}\right)^2}}=1 \tag{2}$$

where c is the light speed in vacuum. Therefore, $\lim_{\ell\omega_{\text{rev}}/\omega_0\to 0}w(z,\omega_0+\ell\omega_{\text{rev}})=w(z,\omega_0)$. Because $w(z,\omega)$ is the only frequency-dependent parameter in the expression for the amplitude of the electric field of $LG_{\ell,p}$ mode, then the limitation

$$\lim_{\ell\omega_{\text{rev}}/\omega_0\to 0}\left|LG_{\ell,p}(r,\theta,z;\omega_0+\ell\omega_{\text{rev}},w_0)\right|$$

$$=\lim_{\ell\omega_{\text{rev}}/\omega_0\to 0}\frac{C_{\ell,p}^{\text{LG}}}{w(z,\omega_0+\ell\omega_{\text{rev}})}\left(\frac{r\sqrt{2}}{w(z,\omega_0+\ell\omega_{\text{rev}})}\right)^{|\ell|}\exp\left(-\frac{r^2}{w^2(z,\omega_0+\ell\omega_{\text{rev}})}\right)L_p^{|\ell|}\left(\frac{2r^2}{w^2(z,\omega_0+\ell\omega_{\text{rev}})}\right)$$

$$=\frac{C_{\ell,p}^{\text{LG}}}{w(z,\omega_0)}\left(\frac{r\sqrt{2}}{w(z,\omega_0)}\right)^{|\ell|}\exp\left(-\frac{r^2}{w^2(z,\omega_0)}\right)L_p^{|\ell|}\left(\frac{2r^2}{w^2(z,\omega_0)}\right)=\left|LG_{\ell,p}(r,\theta,z;\omega_0,w_0)\right| \tag{3}$$

(ii) Limit calculation for the phase profile of the electric field.

We then consider the limit of the phase term of the electric field of an $LG_{\ell,p}$ mode. The phase term can be written as

$$\angle\left(LG_{\ell,p}(r,\theta,z;\omega,w_0)\right)=-k\frac{r^2}{2R(z,\omega)}-kz+\ell\theta+\psi(z,\omega)=-\frac{\omega}{c}\frac{r^2}{2R(z,\omega)}-\frac{\omega}{c}z+\ell\theta+\psi(z,\omega) \tag{4}$$

where the term $\ell\theta$ is a constant. To calculate the limit of the phase term, we separately calculate the limit of three frequency-dependent phase components:

(a) For the part of $\psi(z,\omega)$, we have

$$\Delta phase_1 = \lim_{\frac{\ell\omega_{\mathrm{rev}}}{\omega_0}\to 0} \psi(z,\omega_0+\ell\omega_{\mathrm{rev}}) - \psi(z,\omega_0)$$

$$= \lim_{\frac{\ell\omega_{\mathrm{rev}}}{\omega_0}\to 0} (|\ell|+2p+1)\left(\arctan\left(\frac{z}{z_{\mathrm{R}}(\omega_0+\ell\omega_{\mathrm{rev}},w_0)}\right) - \arctan\left(\frac{z}{z_{\mathrm{R}}(\omega_0,w_0)}\right)\right)$$

$$= \lim_{\frac{\ell\omega_{\mathrm{rev}}}{\omega_0}\to 0} (|\ell|+2p+1)\times\arctan\frac{\frac{z}{z_{\mathrm{R}}(\omega_0+\ell\omega_{\mathrm{rev}},w_0)}\frac{-\ell\omega_{\mathrm{rev}}}{\omega_0}}{1+\frac{z}{z_{\mathrm{R}}(\omega_0+\ell\omega_{\mathrm{rev}},w_0)}\frac{z}{z_{\mathrm{R}}(\omega_0,w_0)}}$$

$$= 0 \qquad (5)$$

(b) For the part of $-\frac{\omega}{c}z$, we have

$$\Delta phase_2 = \lim_{\ell\omega_{\mathrm{rev}}/\omega_0\to 0}\left(-\frac{\omega_0+\ell\omega_{\mathrm{rev}}}{c}z\right)-\left(-\frac{\omega_0}{c}z\right) = \lim_{\ell\omega_{\mathrm{rev}}/\omega_0\to 0} -\frac{\ell\omega_{\mathrm{rev}}}{c}z = -\frac{\ell\omega_{\mathrm{rev}}}{c}z \qquad (6)$$

Note the limit $\ell\omega_{\mathrm{rev}}/\omega_0 \to 0$ is not equivalent to the limit $\ell\omega_{\mathrm{rev}} \to 0$.

(c) For the part of $-\frac{\omega}{c}\frac{r^2}{2R(z,\omega)}$, we have

$$\Delta phase_3 = \lim_{\frac{\ell\omega_{\text{rev}}}{\omega_0}\to 0}\left(-\frac{\omega_0+\ell\omega_{\text{rev}}}{c}\frac{r^2}{2R(z,\omega_0+\ell\omega_{\text{rev}})}\right)-\left(-\frac{\omega_0}{c}\frac{r^2}{2R(z,\omega_0)}\right)$$
$$= \lim_{\frac{\ell\omega_{\text{rev}}}{\omega_0}\to 0} r^2\left(-\frac{1}{\frac{2cz}{\omega_0+\ell\omega_{\text{rev}}}+\frac{\omega_0+\ell\omega_{\text{rev}}}{2cz}{w_0}^4}+\frac{1}{\frac{2cz}{\omega_0}+\frac{\omega_0}{2cz}{w_0}^4}\right)$$
$$= \lim_{\frac{\ell\omega_{\text{rev}}}{\omega_0}\to 0} r^2\frac{-\frac{2cz}{\omega_0+\ell\omega_{\text{rev}}}\frac{\ell\omega_{\text{rev}}}{\omega_0}+\frac{\ell\omega_{\text{rev}}}{2cz}{w_0}^4}{\left(\frac{2cz}{\omega_0+\ell\omega_{\text{rev}}}+\frac{\omega_0+\ell\omega_{\text{rev}}}{2cz}{w_0}^4\right)\left(\frac{2cz}{\omega_0}+\frac{\omega_0}{2cz}{w_0}^4\right)}$$
$$= \lim_{\frac{\ell\omega_{\text{rev}}}{\omega_0}\to 0} r^2\frac{\frac{\ell\omega_{\text{rev}}}{2cz}{w_0}^4}{\left(\frac{2cz}{\omega_0+\ell\omega_{\text{rev}}}+\frac{\omega_0+\ell\omega_{\text{rev}}}{2cz}{w_0}^4\right)\left(\frac{2cz}{\omega_0}+\frac{\omega_0}{2cz}{w_0}^4\right)} \tag{7}$$

Because

$$0 \le \Delta phase_3 \le \lim_{\frac{\ell\omega_{\text{rev}}}{\omega_0}\to 0} r^2\frac{\frac{\ell\omega_{\text{rev}}}{2cz}{w_0}^4}{\left(\frac{\omega_0}{2cz}{w_0}^4\right)^2} = \lim_{\frac{\ell\omega_{\text{rev}}}{\omega_0}\to 0} r^2\frac{2cz}{\omega_0{w_0}^4}\frac{\ell\omega_{\text{rev}}}{\omega_0} = 0 \tag{8}$$

we then get the limit $\Delta phase_3 = 0$.

(iii) Approximation of the complex electric field.

$$\lim_{\frac{\ell\omega_{\text{rev}}}{\omega_0}\to 0}\frac{LG_{\ell,p}(r,\theta,z;\omega_0+\ell\omega_{\text{rev}},w_0)}{LG_{\ell,p}(r,\theta,z;\omega_0,w_0)}$$
$$= \lim_{\frac{\ell\omega_{\text{rev}}}{\omega_0}\to 0}\frac{\left|LG_{\ell,p}(r,\theta,z;\omega_0+\ell\omega_{\text{rev}},w_0)\right|}{\left|LG_{\ell,p}(r,\theta,z;\omega_0,w_0)\right|}$$
$$\times \exp\big(i(\Delta phase_1+\Delta phase_2+\Delta phase_3)\big) = \exp\left(-il\frac{\omega_{\text{rev}}z}{c}\right) \tag{9}$$

Therefore, for $|\ell\omega_{\text{rev}}| \ll \omega_0$, $LG_{\ell,p}(r,\theta,z;\omega_0+\ell\omega_{\text{rev}},w_0) \approx LG_{\ell,p}(r,\theta,z;\omega_0,w_0)\exp(-i\ell\omega_{\text{rev}}z/c)$ at least in the Rayleigh range within the paraxial approximation, where the parameters z and r have limited values. The approximation needs to be further considered when the beam is outside the Rayleigh range.

Supplementary Note 6. Generalization for the generation of rotating-revolving LG beams

Here, we show more analytical details for generalizing our method of generating rotating-revolving $LG_{\bar{\ell},\bar{p}}$ beams with arbitrary $\bar{\ell}$ and $\bar{p}$ values. First, we consider a beam carrying a single $LG_{\bar{\ell},\bar{p}}$ mode on a single angular frequency ω_0 with an electric field of $LG_{\bar{\ell},\bar{p}}^{\text{Cartesian}}(x,y,0;\omega_0,w_0)\exp(i\omega_0 t)$, where

$LG_{\bar{\ell},\bar{p}}^{\text{Cartesian}}(x, y, 0; \omega_0, w_0)$ is the electric field of an $LG_{\bar{\ell},\bar{p}}$ mode in Cartesian coordinates, which equals $LG_{\bar{\ell},\bar{p}}(r, \theta, 0; \omega, w_0)$ with $r = \sqrt{x^2 + y^2}$ and $\theta = \arctan(y/x)$. The beam is then made offset along the negative *x* axis by a distance of R. The electric field $E_0(x, y, 0, t) = \psi(x, y, 0)\exp(i\omega_0 t)$ of the offset beam can be written in two forms: (i) offsetting the electric field of a conventional $LG_{\bar{\ell},\bar{p}}$ beam with its center on the origin; and (ii) expressing the electric field as a combination of multiple $LG_{\ell,p}$ modes [1,2,3]. Therefore,

$$E_0(x, y, 0, t) = LG_{\bar{\ell},\bar{p}}^{\text{Cartesian}}(x + R, y, 0; \omega_0, w_0) \exp(i\omega_0 t) =$$

$$\sum\sum_{\ell,p} C_{\ell,p} LG_{\ell,p}^{\text{Cartesian}}(x, y, 0; \omega_0, w_0)\exp(i\omega_0 t) \tag{10}$$

where $C_{\ell,p} = \iint LG_{\bar{\ell},\bar{p}}^{\text{Cartesian}}(x + R, y, 0; \omega_0, w_0) \left(LG_{\ell,p}^{\text{Cartesian}}(x, y, 0; \omega_0, w_0)\right)^* dxdy$ is a time-independent complex coefficient.

We then consider the case that the above offset beam revolves clockwise around the origin (*i.e.*, the central axis) at a speed of f_{rev} revolutions per second. Namely, the beam dynamically revolves clockwise around the origin to an angle of $\varphi(t) = \omega_{\text{rev}} t = 2\pi f_{\text{rev}} t$. The effect of this motion can be mathematically expressed as a time-dependent coordinate rotation operator $T_{\varphi(t)}$, which is defined as $T_{\varphi(t)}\big(f(r, \theta)\big) = f\big(r, \theta + \varphi(t)\big)$ in cylindrical coordinates and $T_{\varphi(t)}\big(f(x, y)\big) = f(x \cos\varphi(t) - y \sin\varphi(t), x \sin\varphi(t) + y \cos\varphi(t))$ in Cartesian coordinates. After applying the coordinate rotation $T_{\varphi(t)}$ to both sides of Supplementary Eq. (10), we realise two forms of the electric field $E_1(x, y, 0, t)$ of the dynamically rotating-revolving $LG_{\bar{\ell},\bar{p}}$ beam as

$$T_{\varphi(t)}\big(E_0(x, y, 0, t)\big) = E_0(x \cos\varphi(t) - y \sin\varphi(t), x \sin\varphi(t) + y \cos\varphi(t), 0, t) =$$

$$LG_{\bar{\ell},\bar{p}}^{\text{Cartesian}}(x \cos\varphi(t) - y \sin\varphi(t) + R, x \sin\varphi(t) + y \cos\varphi(t), 0; \omega_0, w_0) \exp(i\omega_0 t) \tag{11}$$

$$T_{\varphi(t)}\big(\sum\sum_{\ell,p} C_{\ell,p} LG_{\ell,p}^{\text{Cartesian}}(x, y, 0; \omega_0, w_0)\exp(i\omega_0 t)\big) = T_{\varphi(t)}\big(\sum\sum_{\ell,p} C_{\ell,p} U(r, z =$$

$$0; \omega_0, w_0)\exp(i\ell\theta)\exp(i\omega_0 t)\big) = \sum\sum_{\ell,p} C_{\ell,p} U(r, z = 0; \omega_0, w_0) \exp\Big(i\ell\big(\theta + \varphi(t)\big)\Big) \exp(i\omega_0 t) =$$

$\sum\sum_{\ell,p} C_{\ell,p} U(r, z=0; \omega_0 + \ell\omega_{\text{rev}}, w_0) \exp(i\ell\theta) \exp\left(i(\omega_0 + \ell\omega_{\text{rev}})t\right) = \sum\sum_{\ell,p} C_{\ell,p} LG_{\ell,p}(r, \theta, 0; \omega_0 + \ell\omega_r, w_0) \exp\left(i(\omega_0 + \ell\omega_{\text{rev}})t\right)$ (12)

where the complex term $U(r, z=0; \omega, w_0) = \frac{C_{\ell,p}^{\text{LG}}}{w_0}\left(\frac{r\sqrt{2}}{w_0}\right)^{|\ell|} \exp\left(-\frac{r^2}{{w_0}^2}\right) L_p^{|\ell|}\left(\frac{2r^2}{{w_0}^2}\right)$ is frequency independent so that $U(r, z=0; \omega_0, w_0) = U(r, z=0; \omega_0 + \ell\omega_{\text{rev}}, w_0)$. Supplementary Eq. (12) indicates that a rotating-revolving $LG_{\bar{\ell},\bar{p}}$ beam can be generated by combining multiple frequency comb lines with each carrying multiple $LG_{\ell,p}$ modes. It also shows that the revolution motion of the rotating-revolving beam introduces a frequency shift of $\ell\omega_r$ (*i.e.*, $\omega_0 \to \omega_0 + \ell\omega_{\text{rev}}$) to each $LG_{\ell,p}$ mode [4,5]. Note that Supplementary Eqs. (11) and (12) are equivalent to each other, and there does not appear to be a strict limitation on the $\bar{\ell}$ and $\bar{p}$ values if we select the coefficient $C_{\ell,p}$ to be an integral $\iint \psi(x, y, 0)\left(LG_{\ell,p}(r, \theta, 0; \omega_0, w_0)\right)^* dxdy$ [2,3].

Supplementary Note 7. Diffraction of rotating-revolving *LG* beams

We have shown in the Results Section the diffraction effects on the free-space propagation of a special example (*i.e.*, a rotating-revolving $LG_{3,0}$ beam) in the Article, and it may be interesting to provide an estimation of diffraction effects for the cases with other modal indices $\bar{\ell}$ and $\bar{p}$.

The free-space propagation of a rotating-revolving/conventional $LG_{\bar{\ell},\bar{p}}$ beam can be understood by analyzing the propagation of each $LG_{\ell,p}$ mode carried by the beam. The propagation of a single $LG_{\ell,p}$ mode on a single frequency line is described in Eq. (3) in the Method Section in the Article. Specifically, after propagation from 0 to a distance z, the electric filed $LG_{\ell,p}(r, \theta, 0; \omega, w_0)\exp(i\omega t)$ evolves to $LG_{\ell,p}(r, \theta, z; \omega, w_0)\exp(i\omega t)$ (*i.e.*, *z*-dependent electric field) [6]. Based on this point, if we replace the electric filed at $z = 0$ of each $LG_{\ell,p}$ mode in Supplementary Eq. (10) with the *z*-dependent one, the *z*-dependent electric field of an offset conventional $LG_{\bar{\ell},\bar{p}}$ beam in Supplementary Eq. (10) then takes the form

$E_0(x, y, z, t) = LG_{\bar{\ell},\bar{p}}^{\text{Cartesian}}(x + R, y, z; \omega_0, w_0)\exp(i\omega_0 t) = \sum\sum_{\ell,p} C_{\ell,p} LG_{\ell,p}^{\text{Cartesian}}(x, y, z; \omega_0, w_0)\exp(i\omega_0 t)$ (13)

Furthermore, we consider the propagation of a rotating-revolving $LG_{\bar{\ell},\bar{p}}$ beam. Based on Supplementary Eq. (12), each $LG_{\ell,p}$ mode is carried by a different frequency line on $\omega_0 + \ell\omega_{\text{rev}}$. Similarly, if we replace the electric filed at $z = 0$ of each $LG_{\ell,p}$ mode on the right side in Supplementary Eq. (12) with the z-dependent one, the z-dependent electric field of a rotating-revolving $LG_{\bar{\ell},\bar{p}}$ beam in Supplementary Eq. (12) then takes the form

$$E_1(x, y, z, t) = \sum\sum_{\ell,p} C_{\ell,p} LG_{\ell,p}(r, \theta, z; \omega_0 + \ell\omega_{\text{rev}}, w_0) \exp(i(\omega_0 + \ell\omega_{\text{rev}})t) \quad (14)$$

When $|\ell\omega_{\text{rev}}| \ll \omega_0$, $LG_{\ell,p}(r, \theta, z; \omega_0 + \ell\omega_{\text{rev}}, w_0) \approx LG_{\ell,p}(r, \theta, z; \omega_0, w_0)\exp(-iz/c)$ at least in the Rayleigh range within the paraxial approximation (See Supplementary Note 5 for the approximation details). Such approximation may not hold when the values of z or r reach infinity. Moreover, based on Supplementary Eq. (12), the effect of delaying the electric field of $LG_{\ell,p}(r, \theta, z; \omega_0, w_0)$ by $\exp\left(i\ell\varphi(t, z)\right)$ is equivalent to that of rotating the coordinate by an angle $\varphi(t, z)$ (See Supplementary Note 6 for details). Thus, Supplementary Eq. (14) can be rewritten as

$$E_1(x, y, z, t) \approx \sum\sum_{\ell,p} C_{\ell,p} LG_{\ell,p}(r, \theta, z; \omega_0, w_0) \exp\left(i\ell\omega_{\text{rev}}(t - {}^{z}/_{c})\right) \exp(i\omega_0 t) =$$

$$T_{\varphi(t,z)=\omega_{\text{rev}}(t-{}^{z}/_{c})}\left(\sum\sum_{\ell,p} C_{\ell,p} LG_{\ell,p}(r, \theta, z; \omega_0, w_0) \exp(i\omega_0 t)\right) = T_{\varphi(t,z)=\omega_{\text{rev}}(t-{}^{z}/_{c})}\left(E_0(x, y, z, t)\right) \quad (15)$$

where the coordinate rotation operator $T_{\varphi(t,z)=\omega_{\text{rev}}(t-{}^{z}/_{c})}\left(f(r, \theta)\right) = f\left(r, \theta + \varphi(t, z)\right)$indicates that: after propagation in free space for a distance z at least within the Rayleigh range, the intensity and phase profiles of the generated spatiotemporal beam could be approximated as those of an offset conventional $LG_{\bar{\ell},\bar{p}}$ beam, whose center revolves around the origin by an angle of $\omega_{\text{rev}}(t - {}^{z}/_{c})$. However, such approximation might not hold (i) with further propagation at far-field and (ii) as the frequency difference $\ell\omega_r$ increases. In these two cases, there might be a larger difference between the diffraction effects of the same mode carried by the frequency lines on ω_0 and $\omega_0 + \ell\omega_{\text{rev}}$. As a result, the superposition of multiple modes carried by multiple frequency lines is distorted and thus the mode purity might decrease.

Supplementary References

1. G. Molina-Terriza, J. P. Torres, L. Torner, Management of the angular momentum of light: preparation of photons in multidimensional vector states of angular momentum. *Physical Review Letters* **88**, 013601 (2001).
2. C. Schulze, A. Dudley, D. Flamm, M. Duparré, Andrew Forbes, Measurement of the orbital angular momentum density of light by modal decomposition, *New Journal of Physics* **15**, 073025 (2013).
3. T. Kaiser, D. Flamm, S. Schröter, M. Duparré, Complete modal decomposition for optical fibers using CGH-based correlation filters, *Optics Express* **17**, 9347–9356 (2009).
4. G. Pariente, F. Quéré, Spatio-temporal light springs: extended encoding of orbital angular momentum in ultrashort pulses. *Optics Letters* **40**, 2037-2040 (2015).
5. L. Fang, M. J. Padgett, J. Wang, Sharing a Common Origin Between the Rotational and Linear Doppler Effects, *Laser & Photonics Reviews* **11**, 1700183 (2017).
6. A. M. Yao, M. J. Padgett, Orbital angular momentum: origins, behavior and applications. *Advances in Optics and Photonics* **3**, 161-204 (2011).
