# Supplementary material for: Dynamic spatiotemporal beams that combine two independent and controllable orbital-angular-momenta using multiple optical-frequency-comb lines
Source: Nat Commun. 2020 Aug 14;11:4099. doi: 10.1038/s41467-020-17805-1 (PMC7427811; doi:10.1038/s41467-020-17805-1)
Supplement: Supplementary file 2 — Description of Additional Supplementary Files [file 41467_2020_17805_MOESM2_ESM.pdf]

Description of Additional Supplementary Files

File name: Supplementary Movie 1

Description: The time-variant amplitude and phase profiles of the rotating-revolving $LG_{\bar{\ell},0}$ beams with the value of $\bar{\ell}$ varied from -1 to +3. $\bar{\ell}$ represents the number of 2π phase changes along the azimuthal direction around the beam center.
